# Drought modifies the impacts of soil nutrient heterogeneity on native and alien plant growth in the absence of competition

**DOI:** 10.1093/aobpla/plaf042

**Published:** 2025-08-19

**Authors:** Yin-Ni Wu, Xiao-Yan Na, Lin Huang, Ke-Xin Weng, Wei Xue, Fei-Hai Yu

**Affiliations:** Institute of Wetland Ecology and Clone Ecology / Zhejiang Provincial Key Laboratory of Evolutionary Ecology and Conservation / Zhejiang Key Laboratory for Restoration of Damaged Coastal Ecosystems, Taizhou University, Taizhou 318000, Zhejiang, China; Institute of Wetland Ecology and Clone Ecology / Zhejiang Provincial Key Laboratory of Evolutionary Ecology and Conservation / Zhejiang Key Laboratory for Restoration of Damaged Coastal Ecosystems, Taizhou University, Taizhou 318000, Zhejiang, China; Institute of Wetland Ecology and Clone Ecology / Zhejiang Provincial Key Laboratory of Evolutionary Ecology and Conservation / Zhejiang Key Laboratory for Restoration of Damaged Coastal Ecosystems, Taizhou University, Taizhou 318000, Zhejiang, China; Institute of Wetland Ecology and Clone Ecology / Zhejiang Provincial Key Laboratory of Evolutionary Ecology and Conservation / Zhejiang Key Laboratory for Restoration of Damaged Coastal Ecosystems, Taizhou University, Taizhou 318000, Zhejiang, China; Institute of Wetland Ecology and Clone Ecology / Zhejiang Provincial Key Laboratory of Evolutionary Ecology and Conservation / Zhejiang Key Laboratory for Restoration of Damaged Coastal Ecosystems, Taizhou University, Taizhou 318000, Zhejiang, China; Institute of Wetland Ecology and Clone Ecology / Zhejiang Provincial Key Laboratory of Evolutionary Ecology and Conservation / Zhejiang Key Laboratory for Restoration of Damaged Coastal Ecosystems, Taizhou University, Taizhou 318000, Zhejiang, China

**Keywords:** alien plants, climate change, interspecific competition, spatial soil configuration, soil nutrient heterogeneity

## Abstract

Soil nutrient heterogeneity has generally been shown to benefit alien plants more than native ones. However, whether drought, an important aspect of climate change, alters these effects remains an open question. We used a greenhouse experiment with two alien and two native herbaceous plants. Plants were grown either alone or in a mixture (one alien plant and one native plant) in homogeneous and heterogeneous soils, with or without drought. We found that shoot mass of the native plant *Alternanthera sessilis* and the alien plant *Celosia argentea* were 27.4% and 76.6% lower in heterogeneous soils than homogenous soils, respectively, indicating a negative effect of soil nutrient heterogeneity. However, these negative effects were eliminated when the plants were grown alone in drought conditions. In contrast, soil nutrient heterogeneity, drought, and competition had little effect on the growth of the native plant *Achyranthes bidentata* and the alien plant *Amaranthus retroflexus*. These results suggest that plant species differ in their growth responses to complex environmental changes. These results may have implications for understanding plant invasion outcomes in heterogeneous environments under global climate changes.

## Teaser

A greenhouse study tested how spatial distribution of soil nutrients and drought affect native and alien plants. Two alien and two native herbaceous species were grown alone or together in either evenly mixed (homogeneous) or patchy (heterogeneous) soils, with or without drought. Patchy soils reduced shoot growth of the native plant Alternanthera sessilis and the alien plant Celosia argentea by 27% and 77%, respectively, but these negative effects disappeared when plants were grown alone under drought. In contrast, the native plant Achyranthes bidentata and the alien plant Amaranthus retroflexus were little affected by spatial distribution of soil nutrients, drought, or competition. The findings show that plant species respond differently to the combined effects of spatial distribution of soil nutrients, drought, and competition, which may influence invasion outcomes under climate change.

## Introduction

In nature, soil nutrients are inherently heterogeneously distributed (Xue et al. 2019). A heterogeneous soil often consists of low- and high-nutrient patches of varying sizes (Day et al. 2003, Gazol et al. 2013, Xue et al. 2019). When the size of the nutrient patches within the heterogeneous soils is similar to the size of plant root systems, the plants can selectively place more roots into the high-nutrient soil patches to acquire more resources (Hodge 2004, de Kroon et al. 2005, Gao et al. 2012, Zhou et al. 2023). In this case, soil nutrient heterogeneity can affect growth, physiology, and biomass allocation of individual plants (Birch and Hutchings 1994, Fransen et al. 1998, Wijesinghe et al. 2005, Wang et al. 2022, Pan et al. 2024) and the structures and composition of plant communities (Xue et al. 2019, 2021, 2023, Liu et al. 2021, Vindušková et al. 2023). However, when the size of nutrient patches within the heterogeneous soils is much smaller or larger than the plant root systems, the plants may not show any responses (Wijesinghe and Hutchings 1997, Wijesinghe et al. 2001, Hutchings et al. 2003). Moreover, plants may also fail to respond to soil nutrient heterogeneity when the contrast between the low- and high-nutrient soil patches within the heterogeneous soils is low (Wijesinghe and Hutchings 1999, Hutchings et al. 2003, Wang et al. 2018, 2022, Huang et al. 2023).

Soil nutrient heterogeneity has been frequently shown to benefit alien plants more than native ones, as the alien plants exhibit a greater precision of selective placement of roots (i.e. foraging behaviour) compared with native plants (Davidson et al. 2011, Keser et al. 2014, 2015, Chen et al. 2018). However, the presence of a competitor may alter the effects of soil nutrient heterogeneity on both alien and native plants (Fransen et al. 2001, Gao et al. 2021, Wang et al. 2022). If a competitor can occupy high-nutrient soil patches rapidly and utilize the nutrients more efficiently, it may reduce the benefits that other plants would obtain from soil nutrient heterogeneity (Wang et al. 2022). Therefore, when an alien plant grows with a native plant in a soil consisting of high- and low-nutrient soil patches, positive effects of soil nutrient heterogeneity on the native plant may decrease, while the growth benefits of the alien plant from soil nutrient heterogeneity may not change too much due to competition.

Drought is a major stress and one of the most important stress factors affecting plant growth (Liu et al. 2021, Xue et al. 2023). As a plant may vary in the direction and magnitude of its responses to drought stress and soil nutrient heterogeneity (Liu et al. 2021, Wang et al. 2022, Xue et al. 2023), these two factors may jointly influence plant performance (Maestre and Reynolds 2007). For example, Liu et al. (2021) found that the impacts of drought on plant communities were more pronounced in heterogeneous soils with intermediate patch sizes (12 and 24 cm) compared with those with small or large (0 and 48 cm) patch sizes, due to drought-induced changes in soil properties. Xue et al. (2023) reported that drought enhanced the positive effects of soil nutrient heterogeneity on plant community productivity by reducing the growth of plants in homogeneous soils. Previous studies have found that drought may lead to changes in the dominance of alien plants and alter the biological resistance of native plants to alien plants (Jiménez et al. 2011, Manea et al. 2016, Lu et al. 2022, Carrara et al. 2024). However, how drought may influence the effects of soil nutrient heterogeneity on alien vs. native plants is largely unknown. Moreover, whether the presence of an alien or native competitor can alter the interactive effects of drought and soil nutrient heterogeneity has not been tested.

In this study, we conducted a greenhouse experiment to test how soil nutrient heterogeneity, drought, and the presence of a competitor may influence the growth of two alien plants and two native plants. These plants were grown alone or in a mixture of one alien plant and one native plant in homogeneous or heterogeneous soils under drought or not. Specifically, we asked (i) how does soil nutrient heterogeneity affect the growth of alien and native plants? (ii) Does drought alter the effects of soil nutrient heterogeneity on alien and native plants? (iii) Does the presence of a competitor alter the effects of soil nutrient heterogeneity on alien and native plants?

## Materials and methods

### Plant species

We used two alien plant species, i.e. *Celosia argentea* L. and *Amaranthus retroflexus* L. and two native plant species, i.e. *Alternanthera sessilis* (L.) R. Br. ex DC and *Achyranthes bidentata* Blume in the experiment. All plants are herbaceous species in the Amaranthaceae family. *Celosia argentea* is a taprooted species; it is associated with moist, well-drained soil (https://www.iplant.cn). *Amaranthus retroflexus* can develop a thick taproot but has relatively shallow root systems; it is associated with wet environments and is relatively drought-tolerant (https://www.iplant.cn). *Alternanthera sessilis* exhibits both upright and creeping growth habits and has the ability to develop roots on all stem nodes; it can grow in both wet and dry soils (https://www.iplant.cn). *Achyranthes bidentata* has slender, cylindrical roots and is associated with mild and dry environmental conditions (https://www.iplant.cn). All four species commonly co-occur near ditches, farmlands, swamps and roadsides (https://www.iplant.cn).

We purchased seeds of the four species from a local seed company (Dukourenjia, Ganzhou, China). On 15 July 2022, we sowed seeds of each species in separate germination trays filled with peat (Klasmann-Deilmann, China; N: 0.14 g l^−1^; P: 0.10 g l^−1^; K: 0.18 g l^−1^). After sowing, seeds were covered by evenly adding a 0.5-cm-thick layer of peat to each tray. Water was supplied every day after sowing to keep peat moist and promote seed germination. On 22 July 2022, seedlings of similar size were selected and used in the experiment described below.

### Greenhouse experiment

**Figure 1.**
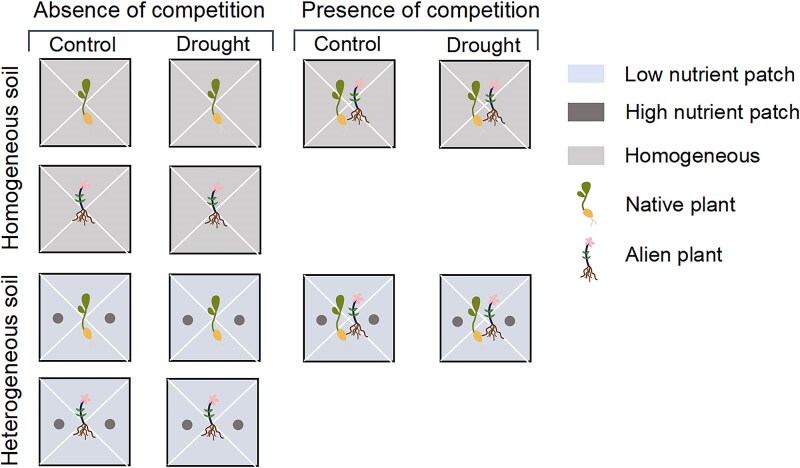
Schematic representation of the experimental design. Plants were grown alone (i.e. absence of competition) or in a mixture of one alien plant and one native plant (i.e. presence of competition) in homogeneous (upper panel) or heterogeneous soils (lower panel) under drought or not.

The experiment was conducted in a greenhouse at the Jiaojiang Campus of Taizhou University in Taizhou, Zhejiang Province, China. The experiment used a factorial design with two levels of soil treatment (homogeneous or heterogeneous), two levels of drought (control or drought), and two levels of competition (absence or presence of interspecific competition between a native and an alien plant). We did not include competition between two native plants or two alien plants, as our primary focus was on how interspecific competition between plants of different invasion status (i.e. native vs. alien) mediates responses to drought and soil nutrient heterogeneity. In the homogeneous soil, we first evenly mixed 2 g of slow-release fertilizer (Everris International BV, The Netherlands; N-P-K = 14-14-14) into a soil (a mixture of peat, field soil and sand at a 2:1:1 volume ratio) and then added the soil to a pot (0.8 l; Fig. 1). In the heterogeneous soil, we first added the soil into a pot, and then we made two holes (1-cm diameter, 4-cm deep) in the centre of two opposite quadrants within the pot (Fig. 1). We added 1 g of slow-release fertilizer to each hole and refilled the hole using the soil in the pot. The slow-release fertilizers are effective for 3–4 months according to the manufacturer’s specifications.

For the treatments without competition, we planted a seedling of a given plant species in the centre of a pot; each plant species was planted in 10 pots (Fig. 1). For treatments with competition, we planted one seedling of an alien plant and one seedling of a native plant 1 cm apart in the centre of a pot. There were four combinations in total (two native × two alien species), and each combination was replicated in two pots. Therefore, there were a total of 96 pots (2 soils × 4 species × 10 pots + 2 soils × 4 interspecific combinations × 2 pots).

All pots were watered every day. Seedlings that died during the first week of the experiment were replaced. On 3 September 2022, half of the pots from each heterogeneity × competition combination were randomly assigned to the drought treatment, and the other half to the control. We added 50 and 150 ml of water every other day to the drought and control pots, respectively. We did not measure the soil water content but did observe that the soil surface was dried and the plants wilted between watering. During the experiment, the mean relative humidity and mean air temperature in the greenhouse were 76.2% and 27.9°C, respectively.

### Harvest and measurements

At the end of the experiment on 15 October 2022, we cut all plants at the soil level and harvested each plant species separately. After clipping, we harvested belowground parts. Since it was not possible to distinguish the roots of different plant species in the competition treatments, we collected all belowground parts together from each pot. All belowground parts were washed through a 0.5-mm sieve. All plant samples were dried at 70°C for at least 48 h.

### Data analysis

We used three-way ANOVAs to examine the effect of soil nutrient heterogeneity (homogeneous vs. heterogeneous), drought (control vs. drought), and competition (absence vs. presence of interspecific competition between a native and an alien plant) on shoot biomass of each plant species. Soil nutrient heterogeneity, drought, competition, and their interactions were included as explanatory factors. As we were specifically interested in the effect of soil nutrient heterogeneity under different drought and competition treatments, a planned contrast was performed to test the difference between the homogeneous and heterogeneous soils under each combination of drought and competition treatment. Post hoc Tukey's HSD tests were also performed to detect differences among different treatments.

We used two-way ANOVAs to examine the effect of soil nutrient heterogeneity and drought on root biomass of each plant species growing in the absence of interspecific competition. In the model, soil nutrient heterogeneity, drought, and their interaction were included as explanatory factors. A planned contrast was performed to test the difference between the homogeneous and heterogeneous soils under each drought treatment. Post hoc Tukey's HSD tests were also performed to detect differences among different treatments.

All analyses were performed using SPSS version 27.0.1. To meet the requirements of normality and homogeneity of variance, root biomass of the native plant *Al. sessilis*, and the alien plant *C. argentea* were transformed to the square root and log, respectively.

### Ethics approval

The species sampled is not endangered or protected.

## Results

**Figure 2.**
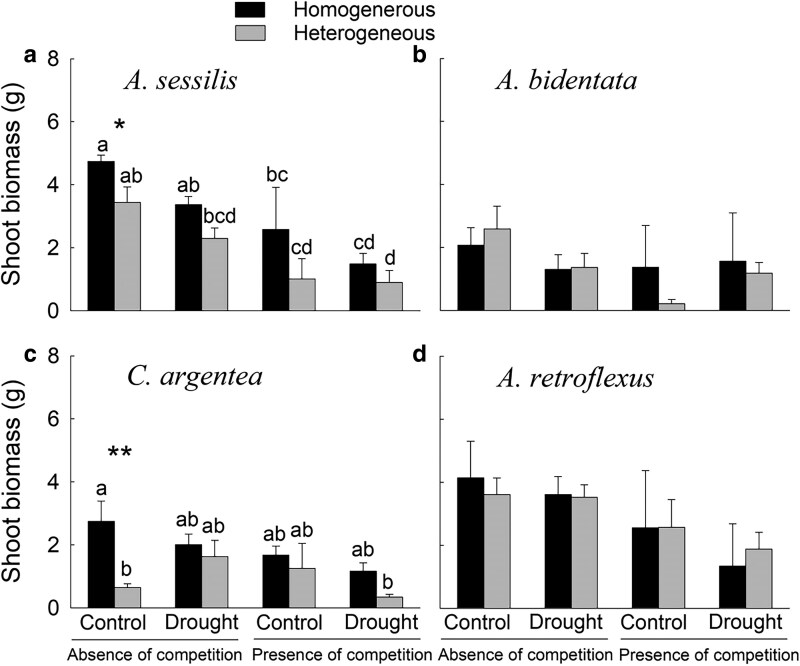
Shoot biomass of the native plants (a) *Alternanthera sessilis* and (b) *Achyranthes bidentata*, and the alien plants (c) *Celosia argentea* and (d) *Amaranthus retroflexus* under different treatments. Different letters (a–d) represent significant differences (*P* < .05; based on post hoc Tukey’s HSD tests) among different treatments. Symbols: *P < .05 and **P < .01 (based on planned contrast).

**Figure 3.**
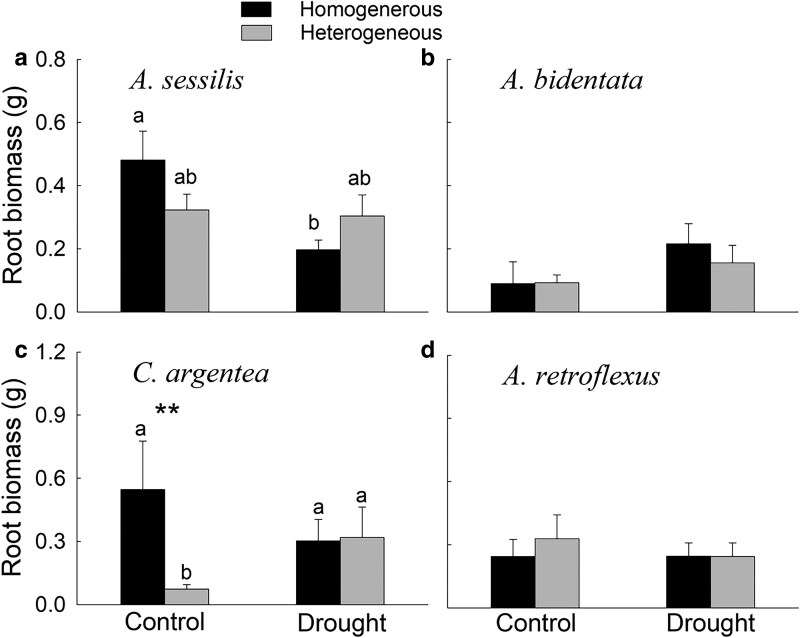
Root biomass of the native plants (a) *Alternanthera sessilis* and (b) *Achyranthes bidentata* and the alien plants (c) *Celosia argentea* and (d) *Amaranthus retroflexus* under different treatments. Different letters (a–b) represent significant differences (*P* < .05; based on post hoc Tukey’s HSD tests) among different treatments. Symbols: **P < .01 (based on planned contrast).

Overall, shoot biomass of the native plant *Al. sessilis* was 27.4% lower in heterogeneous than homogeneous soils (Table 1, *F* = 10.13, *P* = .005; Fig. 2a), but such a difference was not found for root biomass of *Al. sessilis* (Table 2; Fig. 3a). The shoot and root biomass of the alien plant *C. argentea* were 76.6% and 86.7% lower in the heterogeneous than homogenous soils, respectively (Tables 1 and 2, *F* = 5.69, *P* = .027 for shoot biomass and *F* = 5.75, *P* = .029 for root biomass; Figs. 2c and 3c). However, shoot and root biomass of the native plant *A. bidentata* (Tables 1 and 2; Figs. 2b and 3b) and the alien plant *Am. retroflexus* (Tables 1 and 2; Figs. 2d and 3d) did not differ between the heterogeneous and homogeneous soils.

**Table 1.** Results of three-way ANOVAs testing the effects of soil nutrient heterogeneity, drought, and competition on the shoot biomass of the native plants *Alternanthera sessilis* and *Achyranthes bidentate* and the alien plants *Celosia argentea* and *Amaranthus retroflexus*.

Effects	Native plants				Alien plants			
	*Al. sessilis*		*A. bidentata*		*C. argentea*		*Am. retroflexus*	
	*F*_1, 19_	*P*	*F*_1, 16_	*P*	*F*_1, 20_	*P*	*F*_1, 20_	*P*
Heterogeneity (H)	10.13	.**005**	0.21	.651	5.69	.**027**	<0.01	.987
Drought (D)	6.91	.**017**	0.16	.694	0.57	.459	0.85	.369
Competition (C)	30.49	**<**.**001**	2.08	.168	2.76	.112	5.64	.**028**
H × D	0.72	.405	0.02	.879	0.72	.408	0.13	.726
H × C	0.02	.886	1.04	.322	0.64	.434	0.18	.675
D × C	0.85	.369	2.31	.148	1.15	.297	0.22	.647
H × D × C	0.28	.603	0.35	.560	1.86	.188	<0.01	.980

Values are in bold when *P* < .05. Shoot biomass from all pots was included in the analysis.

**Table 2.** Results of two-way ANOVA testing the effects of soil nutrient heterogeneity and drought on the root biomass of the native plants *Alternanthera sessilis* and *Achyranthes bidentate*, and the alien plants *Celosia argentea* and *Amaranthus retroflexus*.

Effects	Native plants				Alien plants			
	*Al. sessilis* ^a^		*A. bidentata*		*C. argentea* ^b^		*Am. retroflexus*	
	*F*_1,16_	*P*	*F*_1,15_	*P*	*F*_1,20_	*P*	*F*_1,20_	*P*
Heterogeneity (H)	0.04	.846	0.29	.604	5.75	.**029**	0.25	.623
Drought (D)	6.67	.**020**	3.03	.110	1.07	.387	0.25	.623
H × D	4.48	.050	0.34	.573	4.02	.062	0.27	.610

Root biomass from pots with single plants was included in the analysis. Values are in bold when *P* < .05.

^a^Data were root-squared transformed.

^b^Data were log-transformed.

Overall, shoot and root biomass of the native plant *Al. sessilis* were 29.0% and 59.1% lower in the drought than in control pots, respectively (Tables 1 and 2, *F* = 6.91, *P* = .017 for shoot biomass and *F* = 6.67, *P* = .020 for root biomass; Figs. 2a and 3a). However, there was no significant main effect of drought on shoot biomass or root biomass of the native plant *A. bidentata* (Tables 1 and 2; Figs. 2b and 3b) or the alien plants *C. argentea* and *Am. retroflexus* (Tables 1 and 2; Figs. 2 and 3).

Overall, shoot biomass of the native plant *Al. sessilis* was 45.5% lower when grown with an alien plant than when grown alone (Table 1, *F* = 30.49, *P* < .001; Fig. 2a). Similarly, shoot biomass of the alien plant *Am. retroflexus* was 38.3% lower when grown with a native plant than when it was grown alone (Table 1, *F* = 5.64, *P* = .028; Fig. 2d). However, competition had no significant effect on shoot biomass of the native plant *A. bidentata* (Table 1; Fig. 2b) or the alien plant *C. argentea* (Table 1; Fig. 2c).

Neither the two-way nor the three-way interactions among soil heterogeneity, drought, and competition had a significant effect on shoot biomass of the native plants *Al. sessilis* and *A. bidentate* (Table 1). However, the planned contrast showed that shoot biomass of *Al. sessilis* was 27.4% lower in the heterogeneous than in the homogeneous soils in the treatment with absence of competition and drought (Fig. 2a). Root biomass of the native plant *Al. sessilis* tended to be lower in the heterogeneous than homogeneous soils under well-watered conditions but higher under drought (Table 2, interaction effect: *P* = .05; Fig. 3a). There was no interaction between drought and nutrient heterogeneity affecting the shoot biomass of the alien plant *C. argentea* and *Am. retroflexus* (Table 1). However, planned contrasts showed that both shoot and root biomass of the alien plant *C. argentea* were significantly lower (by 76.6% and 86.7%, respectively) in heterogeneous compared with homogenous soils in the absence of competition and drought (Figs. 2c and 3c).

## Discussion

Our results showed that soil nutrient heterogeneity reduced growth of the native plant *Al. sessilis* and the alien plant *C. argentea*, and drought eliminated the negative effect of soil nutrient heterogeneity when these plants were grown alone. In contrast, soil nutrient heterogeneity, drought, and competition had little effect on the growth of the native plant *A. bidentata* and the alien plant *Am. retroflexus*. These findings indicate that plant species varied in their growth responses to complex environmental changes, regardless of their native or alien status. Such species-specific responses may have important implications for predicting invasion outcomes in heterogeneous environments under global climate change.

### Negative effects of soil nutrient heterogeneity on both native and alien plant growth

Many studies have shown that soil nutrient heterogeneity can promote the growth of plant species via placement of roots in high-nutrient soil patches (Fransen et al. 1998, Hodge 2004, Wang et al. 2022). However, we found that soil nutrient heterogeneity had negative effects on the shoot and root biomass of both the native plant *Al. sessilis* and the alien plant *C. argentea*. This is consistent with previous studies where both alien and native plants were grown without competition (Keser et al. 2015). The negative effect of soil nutrient heterogeneity observed in our study is likely because the nutrient patches in our study were very small (1-cm diameter) relative to the size of the root system of the plants (Hutchings et al. 2003). In this case, plants may be unable to exploit high-nutrient soil patches efficiently, resulting in poorer growth than in equivalent homogeneous soils. It should be noted that soil nutrient heterogeneity may change during the course of the experiment. We were unable to determine how these changes may have influenced the observed responses, and future studies should take into account the dynamics of soil nutrient heterogeneity.

In contrast, we did not find any effect of soil nutrient heterogeneity on the shoot and root biomass of the native plant *A. bidentata* or the alien plant *Am. retroflexus*. The absence of soil nutrient heterogeneity effects on these two plant species is likely due to their small root systems; the root biomass of these two species was only half as much as that of the other two species (Fig. 3), which showed significant responses to soil nutrient heterogeneity. The native plant *A. bidentata* and the alien plant *Am. retroflexus* may have been unable to respond to the high-nutrient soil patches and thus failed to show a response to soil nutrient heterogeneity (Wijesinghe et al. 2001, Hutchings et al. 2003, Tamme et al. 2016). These results indicate that soil nutrient heterogeneity can influence the growth of both native and alien plant species, but that these effects can vary among different plant species.

### Drought reduced native plant growth but promoted alien plant growth

In this study, drought generally reduced shoot and root growth of the native plant *Al. sessilis* but increased root growth of the alien plant *C. argentea* when it was grown alone in heterogeneous soils. These contrasting results may be due to differences in drought resistance between the native and alien plant species. Previous studies have found that some alien plant species suffered less from drought than native ones did (Catford et al. 2014, Garbowski et al. 2021, Lu et al. 2022). In this study, we found that alien plants may even benefit from drought in heterogeneous environments, particularly in the absence of other stressors. The next step may be to investigate the underlying mechanisms for the contrasting effects of drought on the native plant *Al. sessilis* and the alien plant *C. argentea*. Although plant responses to drought are complex, drought may affect plant performance by reducing nutrient uptake by roots (Maurel and Nacry 2020), increasing transpiration (Farooq et al. 2009), and reducing leaf area where photosynthesis is active (Avramova et al. 2015).

In contrast, the two species, the native plant *A. bidentata* and the alien plant *Am. retroflexus*, that showed no response to soil nutrient heterogeneity also showed no responses to drought. This result indicates that these two species may have better resistance abilities than the other two species to environmental stresses. Plants vary in their abilities to maintain normal physiological and biochemical activities via changes in morphology and physiology, and thus plants may differ in their responses to drought depending on species-specific characteristics (Farooq et al. 2009, Xiong et al. 2020).

### Drought modifies the effects of soil nutrient heterogeneity on plant growth in the absence of competition

Planned contrast showed that, in the absence of competition, the negative effects of soil nutrient heterogeneity on the growth of the native plant *Al. sessilis* and the alien plant *C. argentea*, observed under control conditions, disappeared under drought. For *Al. sessilis*, post hoc comparisons indicated that its root biomass was more negatively affected by drought in homogeneous than in heterogeneous soils (Fig. 3a), and a similar tendency was observed for shoot biomass, although the effect was not statistically significant (Fig. 2a). In contrast, based on post hoc comparisons, *C. argentea* showed increased root growth under drought in heterogeneous soils but not in homogeneous soils (Fig. 3c). A similar pattern was also observed for its shoot biomass, though the effect was not statistically significant (Fig. 2c). These patterns suggest that drought and soil nutrient heterogeneity interact to influence plant growth, although the underlying mechanisms may differ among species. However, given that many pairwise comparisons were not statistically significant, these interactions should be interpreted with caution.

In the presence of competition, the interactive effects of drought and soil nutrient heterogeneity disappeared. This is likely because, under competitive conditions, plant roots may become more crowded, leading to faster depletion of soil water and nutrients. In such conditions, plants may suffer more from drought stress while benefiting less from soil nutrient heterogeneity (Xue et al. 2020, Liu et al. 2021). These results indicate that competition between native and alien plants may alter their responses to drought and soil heterogeneity.

However, we did not detect these interactive effects on the native plant *A. bidentata* and the alien plant *Am. retroflexus*. This may be simply because both species exhibited limited responsiveness to either factor individually, possibly due to greater physiological tolerance or lower plasticity in resource foraging strategies. When species show weak responses to individual environmental factors, significant interaction effects are unlikely to emerge.

## Conclusions

We conclude that spatial heterogeneity of soil nutrients reduced the growth of the native plant *Al. sessilis* and the alien plant *C. argentea*, but had little influence on the native plant *A. bidentata* and the alien plant *Am. retroflexus*. Moreover, drought and the presence of a competitor with contrasting invasion status (i.e. native vs. alien) can alter the impacts of soil nutrient heterogeneity. It should be noted that in this study we only used two native and two alien plants and manipulated soil nutrient heterogeneity at only one spatial scale (i.e. patch size). To test the generality of the results, future studies comparing alien and native species should manipulate soil nutrient heterogeneity at a variety of spatial scales and include a large set of species. Nevertheless, our results highlight the importance of multiple environmental stressors in regulating the growth of native and alien plant performance, which may have important implications for invasive plant control and ecosystem management.

## Data Availability

The data are available at GitHub: https://github.com/dynammor/Biomass-of-native-and-alien-plants.
